# Features of sexual behavior of senior university female students

**DOI:** 10.1192/j.eurpsy.2023.1341

**Published:** 2023-07-19

**Authors:** L. T. Baranskaya, M. Zvychainyi, M. Zagidullina

**Affiliations:** 1 Psychiatry, Psychotherapy and Narcology; 2 Department of Obstetrics and Gynecology, Ural State Medical University, Yekaterinburg, Russian Federation

## Abstract

**Introduction:**

Changes in the attitude of modern society to the intimate sphere - the increasing emancipation of women, their desire for equality with men, including in sexual life, significantly changes the nature of the sexual behavior of young women, which is becoming less dependent on the presence of common myths and stereotypes

**Objectives:**

To identify the features of the sexual behavior of senior university students

**Methods:**

One hundred seventy two senior students of the Ural State Medical University aged 20 to 24 years (average age 21±3.15) attended an anonymous online survey using the Google Forms platform. The author’s questionnaire developed at the Department of Psychiatry, Psychotherapy and Narcology together with the Department of Obstetrics and Gynecology of USMU and included 51 questions.

**Results:**

To the question “Are you brought up in the belief that there is nothing shameful in sex?” answered in the affirmative by 63.0% (108 people) of female students. Most of them 94.0% (162 people) consider it possible to have sexual relations before marriage – “Yes, this corresponds to the norms of modern society.” The age of onset of sexual activity for this sample was 17.5 years. Extreme deviations from the median norm: 14 years - 4.0% (7 people); 23 years old – 2.0% (3 people). To the question “What is your attitude towards representatives of non-traditional sexual orientation?” the answers were distributed as follows: neutral attitude 72.0%, (92 people); 22.0% (28 people) expressed a positive attitude; negative - only - 6.0% (7 people). The study showed that the sexual behavior of young women - university students is determined mainly by intimate and personal attitudes, due to the specifics of society, cultural context, individual and personal characteristics.

**Conclusions:**

Based on the identified trends, the features of the sexual behavior of young women - senior students of the university become more definite and predictable.

**Disclosure of Interest:**

None Declared

